# From design to implementation - The Joint Asia Diabetes Evaluation (JADE) program: A descriptive report of an electronic web-based diabetes management program

**DOI:** 10.1186/1472-6947-10-26

**Published:** 2010-05-13

**Authors:** Gary T Ko, Wing-Yee So, Peter C Tong, Francois Le Coguiec, Debborah Kerr, Greg Lyubomirsky, Beaver Tamesis, Troels Wolthers, Jennifer Nan, Juliana Chan

**Affiliations:** 1 Asia Diabetes Foundation, Flat 4B, Block B, Prince of Wales Hospital, Shatin, Hong Kong SAR, China; 2 Department of Medicine and Therapeutics, The Chinese University of Hong Kong, Prince of Wales Hospital, Hong Kong SAR, China; 3 Merck Sharp & Dohme (MSD), a subsidiary of Merck & Co. Inc., USA

## Abstract

**Background:**

The Joint Asia Diabetes Evaluation (JADE) Program is a web-based program incorporating a comprehensive risk engine, care protocols, and clinical decision support to improve ambulatory diabetes care.

**Methods:**

The JADE Program uses information technology to facilitate healthcare professionals to create a diabetes registry and to deliver an evidence-based care and education protocol tailored to patients' risk profiles. With written informed consent from participating patients and care providers, all data are anonymized and stored in a databank to establish an Asian Diabetes Database for research and publication purpose.

**Results:**

The JADE electronic portal (e-portal: http://www.jade-adf.org) is implemented as a Java application using the Apache web server, the mySQL database and the Cocoon framework. The JADE e-portal comprises a risk engine which predicts 5-year probability of major clinical events based on parameters collected during an annual comprehensive assessment. Based on this risk stratification, the JADE e-portal recommends a care protocol tailored to these risk levels with decision support triggered by various risk factors. Apart from establishing a registry for quality assurance and data tracking, the JADE e-portal also displays trends of risk factor control at each visit to promote doctor-patient dialogues and to empower both parties to make informed decisions.

**Conclusions:**

The JADE Program is a prototype using information technology to facilitate implementation of a comprehensive care model, as recommended by the International Diabetes Federation. It also enables health care teams to record, manage, track and analyze the clinical course and outcomes of people with diabetes.

## Background

In this pandemic of diabetes, Asia has the highest number of affected individuals with a disproportionate increase in the young to middle aged group [1,2]. Although diabetes reduces life expectancy by an average of 10-12 years [3], diabetes and associated complications are highly preventable and treatable [4-7]. Using a comprehensive diabetes registry established since 1995, our group has developed a series of risk equations [8-14] to predict all-cause death and cardiovascular-renal outcomes in Chinese type 2 diabetic patients. Our group and others have also reported 50-70% risk reduction in these clinical outcomes amongst patients receiving protocol-driven care delivered by a multidisciplinary team compared to usual care [15-20].

In 2006, we developed a web-based Joint Asia Diabetes Evaluation (JADE) Program to combine the concepts of risk stratification and protocol-driven care to translate evidence to practice using information technology. In the past years, various computerized systems have been developed to facilitate management of diabetes or cardiovascular disease [21-26]. Most of the systems were data registries though some of them incorporated clinical practice guidelines and/or performance feedback. However, a comprehensive and easily accessible electronic system which integrates primary and secondary care with decision support, risk stratification and interactive feedback, as recommended by the International Diabetes Federation [27], is still lacking. In this program, we used an electronic portal (e-portal) to facilitate health care team to implement evidence-based care protocol and empower patients to improve self management. The administration of the Program is supported by a multidisciplinary team including a part-time endocrinologist, programmer, project coordinator. The e-portal is designed specifically to cater the needs of Asian countries in terms of language and risk equations which stratify patients into 4 care levels to guide clinical management, although these features can be easily adapted for other populations. The e-portal also generates concise graphic reports for both patients and physicians highlighting trends of "ABC" targets (i.e. glycated hemoglobin [HbA_1c_], blood pressure [BP] and low-density lipoprotein cholesterol [LDL-C]) to facilitate individualizing treatment. We have reported the internal validation of the JADE Risk Engine [28] using a registry consisting of 7534 type 2 diabetic patients. Herein, we report the technical details of the design and implementation of this web-based program.

## Results

The JADE portal is available to registered users since November 2007. In this early phase of implementation, we have limited the number of users by invitation to gain feedback, assess need of technical support and refine the Program. Eleven Key Opinion leaders from 8 countries or areas in Asia (China, Hong Kong, Taiwan, Singapore, Malaysia, Thailand, Philippines and Korea) were invited to form a Steering Committee. These steering committee members recommended appropriate clinicians in their countries to the JADE Portal Support Office (PSO). After registration, these clinicians were granted a log-in account with password.

### JADE e-portal http://www.jade-adf.org

**Figure 1:**
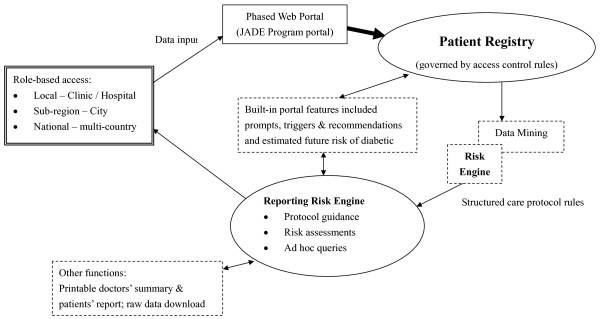
**Logistics and components of the web-based electronic portal of the JADE Program**.

Figure 1 summarizes the key features of the JADE e-portal as a Java application using the Apache web server, the mySQL database and the Cocoon framework. The user input is validated both on the server-side and client-side, which is aligned with industry best coding practices. The validation technique prevents attackers from inserting potentially harmful syntax into the web application, which could then potentially be sent to the application server or backend database residing inside the network infrastructure. The system was developed in a secure manner and externally assessed by Cybertrust Inc. Through SQL injection, cross-site scripting and parameter manipulation, it was unable to gain session hijacking and authentication bypass.

The JADE e-portal includes a validated risk engine for risk stratification based on data collected during the annual comprehensive assessment. Based on the patient's risk level, a care protocol is recommended with clinical decision and self management support. The e-portal also provides user-friendly templates to guide users to collect relevant clinical data during the annual comprehensive assessment visit including eye and foot examination, blood and urine tests, medications, major medical events and quality of life. The British National Formulary was used as a framework to classify medications for data capture and future analysis.

**Figure 2:**
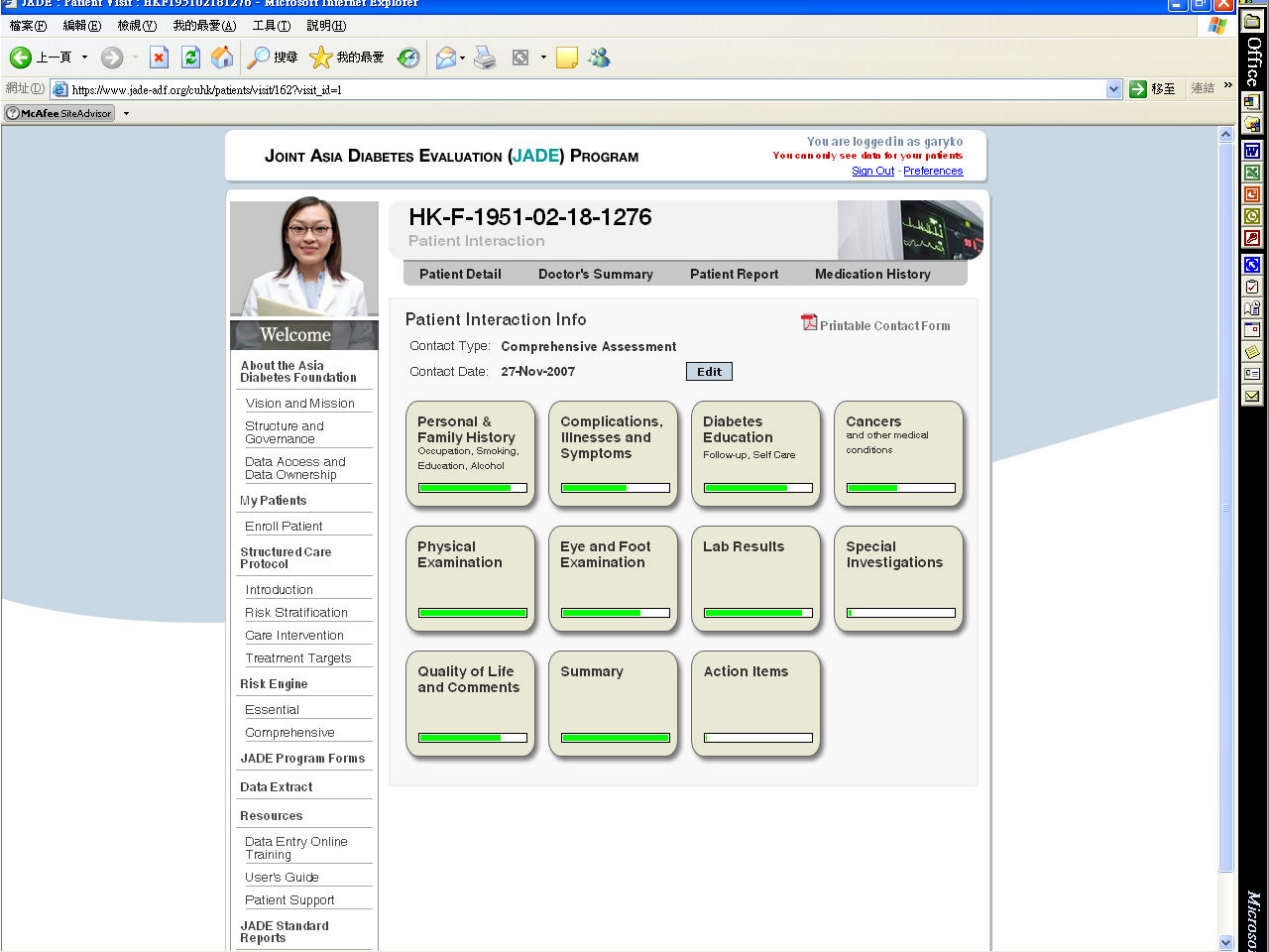
**The JADE electronic portal provides templates to guide users to collect relevant data during the annual comprehensive assessment**.

At each review visit, key parameters as recommended by international guidelines are captured to document clinical progress. These include BP, body weight, risk factor control (e.g. HbA_1c_, lipid, renal function, albuminuria), self care, hypoglycemia and admissions since last visit (Figure 2, showing the comprehensive assessment webpage). These data can be collected on line at point-of-care, or offline using paper format followed by date entry to the e-portal at a later stage, depending on the clinic set up and operation. All patients are given unique identification codes and each visit is dated to avoid double entry. All data are locked 12 weeks after the input to prevent 'back-date' data manipulation. However, through request to the PSO with clear explanation, unlocking of the relevant data for editing or deletion is possible. The timing of 12 weeks for data locking was selected since this was the average turnover time for return of laboratory results and data input in various Asian countries.

**Figure 3:**
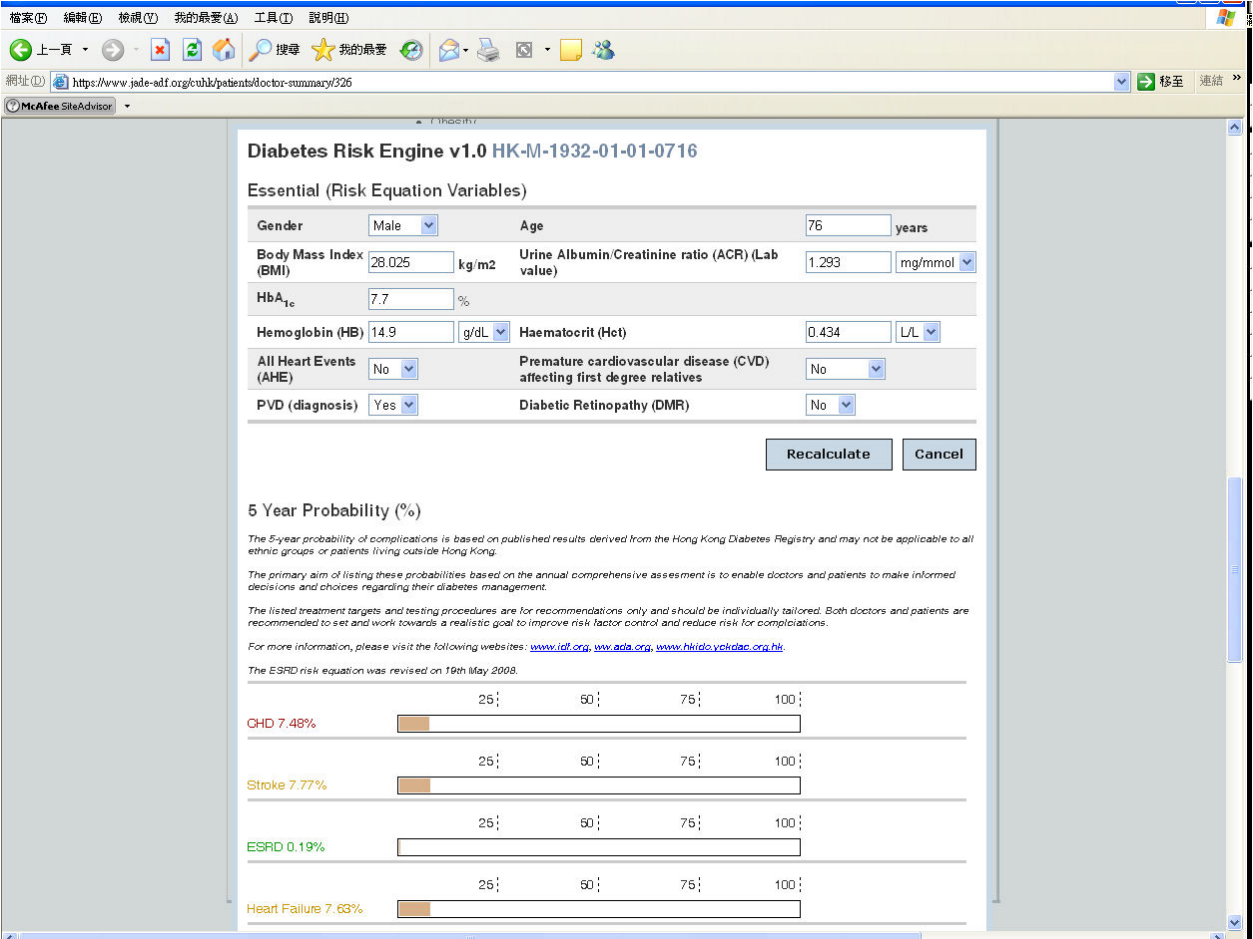
**The JADE Risk Engine estimates the 5-year probability of major clinical events**.

**Figure 4:**
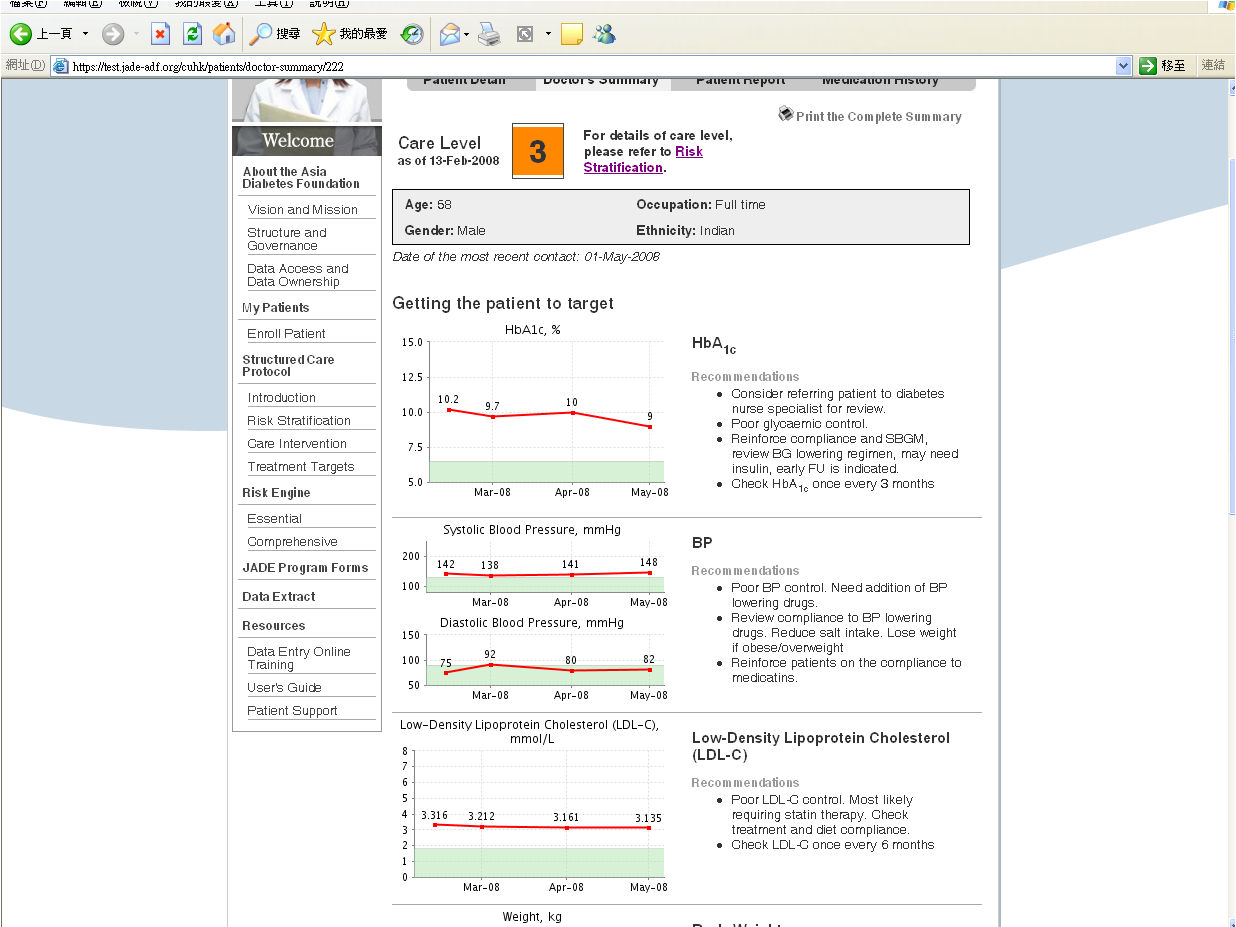
**The JADE e-portal displays the risk level of the patients, supplemented by decision and self management support triggered by various risk factors which can be printed in 5 Asian languages**.

Based on results estimated by the JADE Risk Engine, the e-portal displays the 5-year probability of major clinical events which can be adjusted by changing values of modifiable risk factors to promote discussions between patients and care providers (Figure 3). Data collected at each review visit are displayed to show the trends of control of modifiable risk factors including BP, HbA_1c_, LDL-C and body weight. General recommendations can be triggered by predefined levels of risk factors to prompt care providers and patients to take appropriate actions. Printable reports showing risk predictions, trends of risk factor control and practice tips can be generated for care providers (in English) and patients (in 5 different Asian languages i.e. English, Thai, Korean, Malay and Chinese [both traditional and simplified Chinese]) for record purpose (Figure 4). Furthermore, the portal provides matrixes to help doctors monitor patients' levels of adherence to care processes (e.g. annual assessment, review visits, education sessions, laboratory tests) and self management as well as their status of attainment of treatment targets. These targets can be modified depending on the evolution of international healthcare standards.

Up to 1^st ^January 2010, 10,164 patients from Hong Kong, Taiwan, Singapore, Thailand, Philippines, Korea, India and Brunei have joined the JADE Program. The Program is scheduled to be launched in Mainland China in mid 2010.

## Discussion

Although optimal management of risk factors and treatment to targets can substantially reduce risk of diabetic complications [4-6,29-32], there are multiple barriers at the system, care providers and patients' levels to translate these evidence to clinical practice. These include the complex nature of care protocols, need to interpret large amount of interrelated clinical information, demands on patients' self discipline as well as lack of time and resources for counseling and reinforcement of compliance. In most national or international surveys, standards of diabetes care remain suboptimal often due to low adherence to treatment guidelines and clinical inertia on the part of care providers and low levels of compliance with drugs and self care by patients [33-37].

Motivated by improved risk factor control and clinical outcomes in patients randomized to clinical trials [18,38], our group and others have demonstrated that delivery of protocol-driven care using a multidisciplinary team with particular focus on risk stratification, periodic assessments, treatment to target and reinforcement of treatment compliance including use of telephone counseling can reduce risk of all-cause death and cardio-renal outcomes by 50-70% [6,17-19,33,38-41].

Using these prototypes of care models as templates and through private-public partnerships, we used *state of the art *information technology to develop the JADE Program as a regional quality improvement program. We also used this virtual platform to promote collective learning and sharing of best practices based on evidence pertinent to Asian populations. The latter include the lower cutoff values for body mass index and waist circumference to define overweight/obesity as well as the high predictive values of renal function, albuminuria and low blood hemoglobin for cardiovascular disease. The JADE Program also displays trend lines with prompts and recommendations to reinforce key messages and empower patients and care providers to make informed decisions. Most importantly, health care teams can use the JADE e-portal to create its own diabetes registry to detect defaults, monitor clinical progress and track key performance indexes (e.g. attainment of treatment targets) of their patients for continuous quality improvement.

Supported by the JADE Project team and the Steering Committee, the JADE Program also provides a virtual platform to promote collaborative research to address epidemiological and therapeutic questions pertinent to Asian populations. The establishment of the Asia Diabetes Database using the JADE e-portal aims to refine the accuracy and applicability of various risk equations to different Asian populations. By recording essential clinical data on an ongoing basis as recommended by international guidelines, the impact of adherence to medications and care processes by patients (e.g. self blood glucose monitoring) and care providers (e.g. annual assessment and laboratory investigations); use of non-pharmacological (e.g. diabetes education, lifestyle modification) and pharmacological interventions as well as their interactions with various phenotypes and secular changes on clinical outcomes can be monitored and analyzed [42-44].

### Limitations

The JADE Program is designed for diabetes and associated conditions and complications. It is disease specific that limits its application in non-diabetic subjects. However, diabetes is common and complex, for which a specific and comprehensive management tool is indicated. In addition to using graphic reports to motivate self management, there are rooms for further improvement such as education on appropriate drug use and medication adherence. Continuous review of the e-portal in terms of user-acceptability and recommendations based on evolving evidence is the major task of the JADE Project team. Of note, in order to fully utilize the features of the JADE e-portal, there is a need to modify the clinic practice to deploy trained non-medical personnel, e.g. nurses and physician assistants (e.g. high school or university graduates) to perform clinical assessments, enter data and remind doctors and patients to adhere to recommended treatments or procedures. Currently the JADE Project team is organizing a 1-2 day training program to share experience with health care teams on how to reorganize the clinic setting in order to use the e-portal more effectively using a multidisciplinary care model. Given the complex nature of diabetes and pluralistic needs of those affected, in spite of the *state of the art *information technology, a competent and caring clinical team remains the essence of quality diabetes care [21,45,46].

## Conclusions

We combined the concept of risk stratification and multidisciplinary care to use information technology to facilitate implementation of evidence-based diabetes care protocols. Using a multidisciplinary approach, doctors can use the JADE e-portal to reorganize the process of care delivery and establish a diabetes registry to manage, track and analyze the large amount of clinical information to improve decision making. On a research front, the JADE Program provides a virtual platform to collect epidemiological data and implement disease management programs to improve our understanding of the natural history of diabetes and evaluate effectiveness of various intervention in a pragmatic, naturalistic and scientific setting.

## Methods

### Objective and Design of the JADE Program

The objective of the JADE Program is to use information technology to facilitate health care team to deliver high quality diabetes care so as to reduce complication rates and improve self care. Figure 1 summarizes the logistics and components of the JADE e-portal including role-based access rules, a risk engine, a diabetes registry and care protocols with reporting function. All enrolled patients gave written informed consent to submit their anonymized data for joint analysis. These data include clinical and demographic information and laboratory investigations during regular comprehensive assessments and regular follow-up visits. All participating doctors gave written informed consent and indicated their understanding of the rationale, purpose and implementation process of the JADE Program. This JADE e-portal is available on the internet and targeted at both specialists or primary care doctors who look after diabetic patients in solo practice, public health institutions, health maintenance organizations (HMO) or non-government organizations (NGO).

### Risk Stratification and Care Management Level

The risk stratification program was based on 4 clinical parameters: 1) stratification parameters such as smoking status; 2) estimated glomerular filtration rates (eGFR); 3) risk scores derived from the Hong Kong Diabetes Registry Risk Equations; and 4) the existence of any known cardiovascular-renal complications (table 1) [8-14,27,28,47-50]. The internal validation of this risk stratification program has been reported [28]. In brief, based on these 4 sets of parameters collected during an annual comprehensive assessment, patients can be categorized into one of the 4 care levels: 1) Very High Risk (VHR) group with clinically evident cardiovascular-renal complications; 2) High Risk (HR) group with 3 or more stratification parameters, or values above the high specificity cutoff for any one of the risk scores, or eGFR <60 ml/min/1.73 m^2^; 3) Medium Risk (MR) group with 2 stratification parameters, and/or values above the high sensitivity cutoff but lower than the high specificity cutoff for any of the risk scores, and/or eGFR between 60 and 90 ml/min/1.73 m^2^; and 4) Low Risk (LR) group with one or fewer stratification parameter, and values below the high sensitivity cutoff for all risk scores, and eGFR ≥ 90 ml/min/1.73 m^2^.

**Table 1:** Parameters used in the Joint Asia Diabetes Evaluation (JADE) electronic portal to derive the Care Management Level in Type 2 Diabetic Patients.

Derivation parameters	Details
1. Clinically evident cardiovascular-renal complications	History of stroke, coronary heart disease, non-fatal heart failure, peripheral vascular disease, end stage renal disease (dialysis or eGFR <15 ml/min/1.73 m^2^)

2. Risk stratification parameters	Current or ex smoker BMI ≥ 27.5 kg/m^2 ^or waist circumference ≥ 80 cm in women or ≥ 90 cm in men Blood pressure >130/80 mmHg or treatment with anti-hypertensive drugs LDL-C >2.5 mmol/l TG ≥ 2.3 mmol/l &/or HDL-C <1 mmol/l Treatment with lipid regulating drugs if LDL-C ≤ 2.5 mmol/l and TG <2.3 mmol/l and HDL-C ≥ 1.0 mmol/l Spot morning urine for ACR >3.5 mg/mmol in women or >2.5 mg/mmol in men Foot at risk defined by 2 of 3 of the following: sensory neuropathy, skin changes (e.g. fungal infection, dry skin) or deformities (e.g. claw feet or hallux deformities) Retinopathy HbA_1c _≥ 8%

3. Hong Kong Diabetes Registry Risk Scores	High sensitivity and specificity cutoff points for various clinical endpoints (stroke, coronary heart disease, end stage renal disease and heart failure)

4. Renal function	eGFR by the MDRD equation

eGFR, Estimated glomerular filtration rate; BMI, body mass index; LDL-C and HDL-C, low- and high-density lipoprotein cholesterol; TG, triglyceride; ACR, albumin creatinine ratio; HbA_1c_, glycated hemoglobin; MDRD, Modification of Diet for Renal Disease (ref. [40])

Based on these risk levels, a care protocol with predefined schedules and decision support is recommended [28]. Depending on the risk level, the e-portal will recommend intervals between review visits, laboratory tests and complication assessments. The risk level is estimated based on a comprehensive set of data collected at presentation and then 12-24 monthly thereafter for quality assurance and re-evaluation of risk level. Hence, the risk level, and corresponding management plan, may change according to the clinical status to promote cost-effective use of finite resources. Using charts and trend lines, physicians are encouraged to individualize therapies based on patients' profile and trend of 'ABC' targets to promote self care and reduce clinical inertia. The value of early intervention is now clearly confirmed by the legacy effect of attaining glycemic control early to reduce risk of future complications [7]. Although similar legacy effect has not been confirmed for dyslipidemia and blood pressure [51], optimal treatment of these conditions has been shown to reduce cardio-renal complications in both primary and secondary prevention [6].

### Self management support

The JADE e-portal generates patients' report in simple layman terms and displays the trends of HbA_1c_, BP and LDL-C in diagrammatic forms. There are also prompts and practice tips on lifestyle and use of medication to achieve recommended treatment targets (i.e. HbA_1c _<7%, BP <130/80 mmHg, LDL-C <2.6 mmol/L) [27,28,49,50]. At each visit, the e-portal re-estimates the 5-year probability of cardio-renal complications using the most updated clinical information of the patient, notably, risk factors which are modifiable. To this end, there is evidence suggesting that awareness of complications, health literacy [52] self efficacy and expectation of improved clinical outcomes [53] are associated with improved metabolic control and self care in diabetic patients. While the JADE Program uses care level and risk prediction to empower patients, additional input by the health team will be needed, e.g. referral to dietitians or nurse educators or drug titration or change of medications.

### Governance, technical support and data security

The JADE Program is governed by the Board of Directors of the Asia Diabetes Foundation (ADF) which is a non-profit making organization incorporated under the Chinese University of Hong Kong (CUHK) Foundation. It is a charitable body approved by the Tax and Revenue Department of the Hong Kong Government with a mission to promote awareness and facilitate academic research to improve chronic disease management including diabetes. Hitherto, access to the JADE e-portal is by invitation and free of charge. The ownership and liability of the Program as well as all collected data and subsequent deliverables including publications belong to the ADF.

On an academic front, the JADE Steering Committee consists of leading diabetologists from Asia who provide overall direction and monitor progress of the Program. Members of the JADE Steering Committee also serve as local champions to promote the adoption of the JADE Program in their country. The JADE Project team consists of a medical director working closely with the Information Technology Team, PSO and a supporting team in each participating country.

To limit the impact of potential exposure or leakage of patient information, the JADE Program does not store any identifying patient information electronically. No name or national identity number is captured and a case-specific code (ADF code) is generated for each enrolled patient. Only the physician has the information to identify the patient given a specific ADF code. These case sensitive codes are known to the patients and all report forms generated electronically or in paper form, are kept by the physicians or in the case records as appropriate. Registered users, who are usually the doctor in charge of the clinic, can grant access rights to other clinic staff. These assistant personnel need to provide written consent and abide to the terms of reference of ADF before they can use the e-portal and have access to these anonymized data which can be decoded using the patient log kept at the clinic. At the time of writing, patients are given printed reports which summarize their risk levels, 5-year risk of major events and key clinical parameters. They do not have access to their raw data electronically, although this feature can be incorporated at a later stage, depending on the uptake of the Program.

All data are given a 'reasonable' range for acceptance and will generate a prompt if data are above or below the range. The validity of all clinical information is checked periodically by a monitoring team designated by the JADE Project team to ensure the quality and integrity of these data for analysis purpose. While the monitor cannot change any data, he/she can flag reminders to the local supporting team when suspicious data is encountered such as large discrepancy in body mass index and waist circumference. For errors noted after the data has been locked, the corresponding user can request data unlocking for amendment. All editing activities are tracked and recorded by the PSO.

Apart from using anonymized data, the JADE e-portal also adopts the strictest security industry practices. These include end-to-end data encryption of the grade used by most online banking sites, user authentication, information access control mechanisms, and dedicated server hosting with physical access control to the facilities. In July 2007, Cybertrust Inc., an established and specialized security company, was commissioned to perform an independent security assessment of the application of the JADE e-portal. Cybertrust conducted both black box and white box security testing of the application, and verified that the JADE e-portal performed reliably and securely when tested with automated and manual attack techniques.

### Institutional board review and ethics approval

The establishment of the Asia Diabetes Database using the JADE e-portal was approved by the Clinical Research Ethics Committee of the Chinese University of Hong Kong. Implementation of the JADE Program was also approved by the relevant institutional review board in individual countries through submission by the Steering Committee members. All patients were given detailed information sheet on the background, purpose and processes of the JADE Program. Written consent was obtained from each participant to undergo comprehensive assessment at enrolment and at regular intervals thereafter. Participating doctors and care providers also gave written informed consent indicating their understanding and willingness to promulgate the vision and mission of the JADE Program to improve ambulatory diabetes care.

### Portal Support Office (PSO)

The Information Technology team based at the JADE PSO supports all program-related technical and logistic matters encountered by the users. The PSO organizes meetings with the JADE supporting teams in different countries on a regular basis to collect feedback and explain changes to the e-portal, as appropriate. All users are provided a manual of frequently asked question (FAQ) about the JADE e-portal. The PSO also provides information on evidence-based management plan of updated versions, scheduled maintenance, bug reports and technical tips. The service commitment in supporting on-line enquiry of the PSO include response time in 2 working days and resolution time in 2 to 7 days depending on complexity of the enquiries.

## Authors' contributions

GK, WS, PT and JC conceptualised and designed the project. FC, DK and GL provided technical input throughout the project. GK and JC drafted the manuscript. WS, PT, GL, BT, TW and JN contributed to the intellectual content of the manuscript. All authors read and approved the final manuscript.

## Pre-publication history

The pre-publication history for this paper can be accessed here:

http://www.biomedcentral.com/1472-6947/10/26/prepub
